# New Approaches for Enhancement of the Efficacy of Mesenchymal Stem Cell-Derived Exosomes in Cardiovascular Diseases

**DOI:** 10.1007/s13770-022-00469-x

**Published:** 2022-07-22

**Authors:** Lamiaa Ahmed, Khaled Al-Massri

**Affiliations:** 1 Department of Pharmacology and Toxicology, Faculty of Pharmacy, Cairo University, Kasr El Aini St., Cairo, 11562 Egypt; 2 Department of Pharmacy and Biotechnology, Faculty of Medicine and Health Sciences, University of Palestine, Gaza, Palestine

**Keywords:** Cardiovascular diseases, Exosomes, Mesenchymal stem cells

## Abstract

Cardiovascular diseases (CVDs) remain a major health concern worldwide, where mesenchymal stem cells (MSCs) therapy gives great promise in their management through their regenerative and paracrine actions. In recent years, many studies have shifted from the use of transplanted stem cells to their secreted exosomes for the management of various CVDs and cardiovascular-related diseases including atherosclerosis, stroke, myocardial infarction, heart failure, peripheral arterial diseases, and pulmonary hypertension. In different models, MSC-derived exosomes have shown beneficial outcomes similar to cell therapy concerning regenerative and neovascular actions in addition to their anti-apoptotic, anti-remodeling, and anti-inflammatory actions. Compared with their parent cells, exosomes have also demonstrated several advantages, including lower immunogenicity and no risk of tumor formation. However, the maintenance of stability and efficacy of exosomes after *in vivo* transplantation is still a major concern in their clinical application. Recently, new approaches have been developed to enhance their efficacy and stability including their preconditioning before transplantation, use of genetically modified MSC-derived exosomes, or their utilization as a targeted drug delivery system. Herein, we summarized the use of MSC-derived exosomes as therapies in different CVDs in addition to recent advances for the enhancement of their efficacy in these conditions.

**Graphical abstract:**

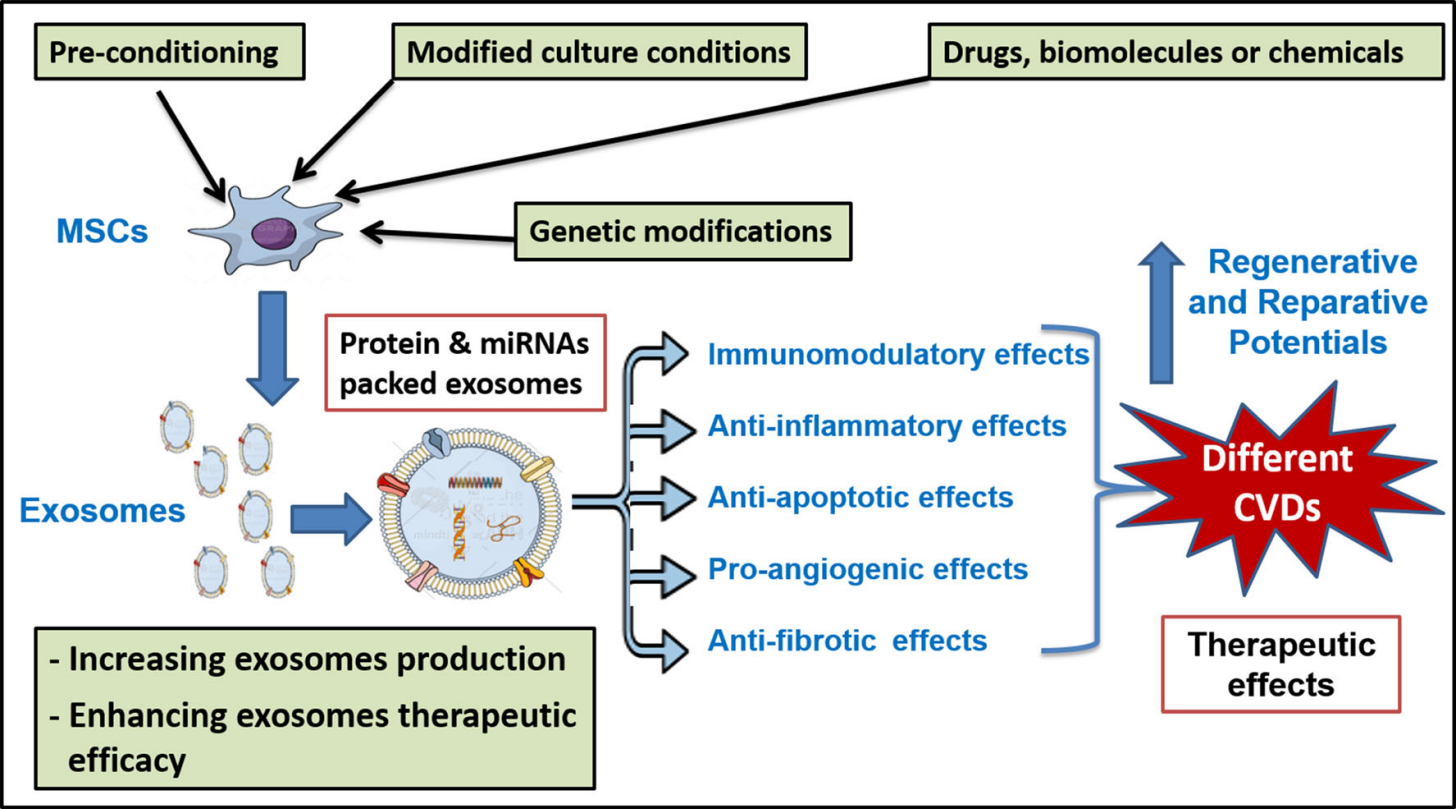

## Introduction

Mesenchymal stem cells (MSCs) are multipotent adult progenitor cells with differentiation and self-renewal capabilities. They could be isolated from various sources, including adipose tissues, umbilical cord blood, bone marrow (BM) [1, 2]. MSCs have been revealed as a promising candidate in different cardiovascular diseases (CVDs) [3–5]. The therapeutic potential of MSCs has been proposed to depend partly on their capacity to secrete extracellular vesicles (EVs) and other paracrine factors [6–9].

EVs consist mainly of exosomes, microvesicles, and apoptotic bodies. MSC-derived exosomes display biological functions similar to MSCs, contributing to tissue regeneration by enclosing and conveying various cytokines (e.g., interleukin (IL)-6 and IL-10) [10, 11], growth factors (e.g., transforming growth factor beta and hepatocyte growth factor (HGF)) [12, 13], signaling lipids [14], mRNAs (e.g., insulin-like growth factor 1 receptor (IGF-1R) [15], and regulatory miRNAs (e.g., miRNA-21 and miRNA-133b) [16, 17]. Exosomes have demonstrated several advantages over pure cell treatments, including lower immunogenicity and no risk of tumor formation [18].

**Fig. 1:**
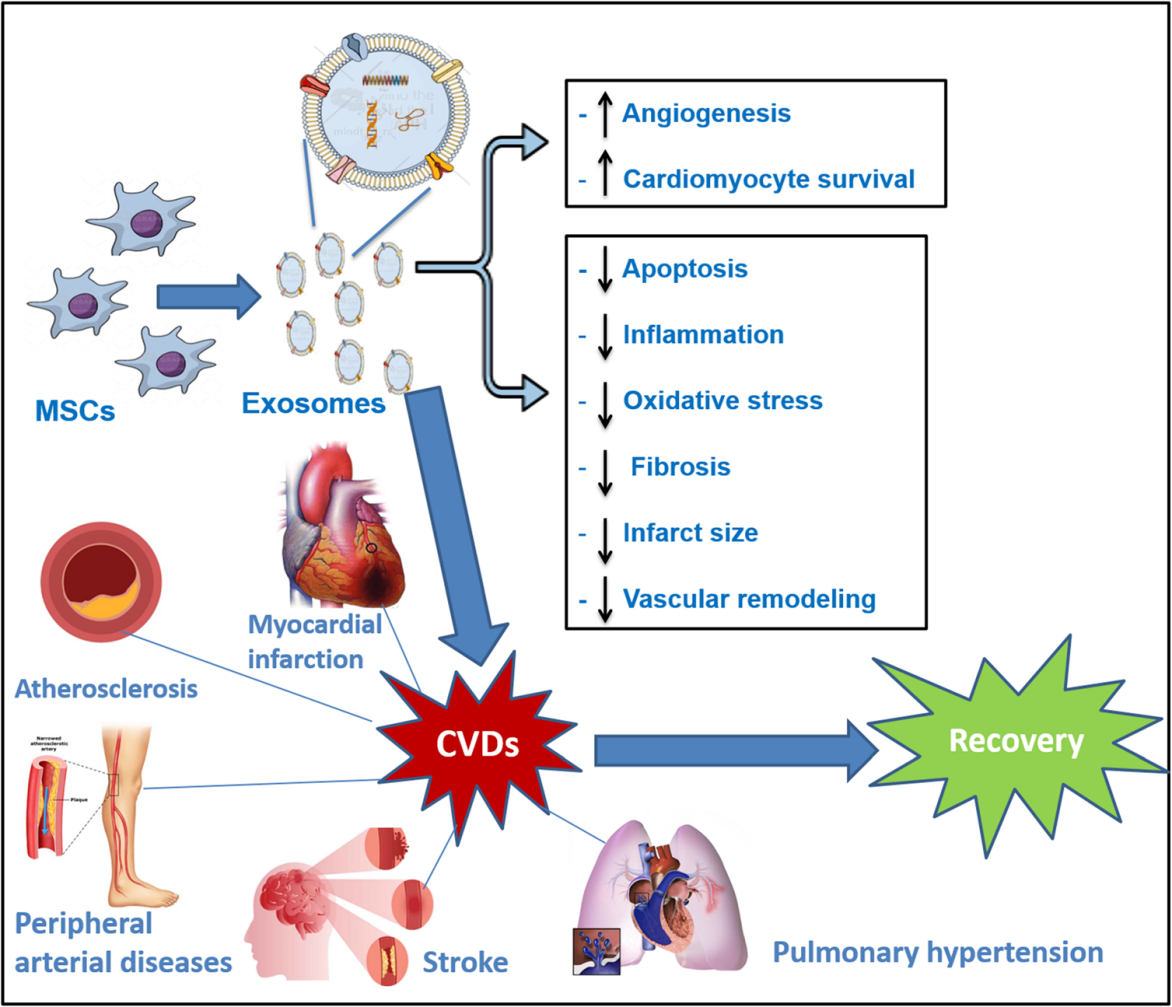
MSC-derived exosomes as a potential therapy for different CVDs. Exosomes deliver nucleic acids (DNAs/RNAs) and proteins to the damaged tissues and consequently exert their protective effects through different mechanisms

Recent studies have shown that MSC-derived exosomes could represent a novel therapeutic approach for the management of different CVDs and cardiovascular-related diseases (Fig. 1). Essentially, exosomes secreted by MSCs have revealed significant roles in cytoprotection, stimulation of angiogenesis, inhibition of cardiac fibroblast development, and modulation of macrophage infiltration in the infarcted region [19, 20].

Moreover, several preclinical studies have confirmed that MSC-derived exosomes reduced the infarct size, leading to the recovery of blood flow and improvement of post-myocardial infarction cardiac function [21–23]. Despite these significant benefits, the clinical application of exosomes reveals several limitations including the maintenance of efficacy and stability of exosomes over time after *in vivo* transplantation [24]. Thus, new approaches have been continuously developed to enhance their efficacy and stability including their preconditioning before transplantation, use of genetically modified MSC-derived exosomes, or their utilization as a targeted drug delivery system [25].

## MSC-derived exosomes and their origin

Since their first successful transplantation in 1968 [26], cell therapies have been used as an alternative therapy in various diseases, particularly CVDs [27–29]. Stem cell-based therapies could repair the damaged cardiomyocytes and subsequently improve cardiac function in several preclinical and some clinical trials [30]. However, their clinical applications have limitations, including low survival and engraftment, immune rejection, and risk of tumorigenic potential [31].

Interestingly, stem cells can repair injured tissues *via* paracrine actions through their derived exosomes [32]. Exosomes are a homogenous population of EVs that originate from endosomes and are released by parent cells extracellularly. Exosomes are nanosized vesicles with a diameter of 30–100 nm; thus, they differ from other EVs that originate from the plasma membrane with a diameter of 100–1000 nm. Exosomes are novel mediators of cell-to-cell signaling and communication through the transfer of mRNA, regulatory miRNAs, cytokines, peptides, and signaling lipids leading to information transfer and coordinative function between cells [33].

Exosomes, as nanosized EVs, are distributed through a vast complex of body fluids, which represents a challenge in their isolation. Several types of exosome separation strategies have been reported till now, including ultrafiltration, ultra-speed centrifugation, charge neutralization-based polymer precipitation, immunoaffinity capture, size-exclusion chromatograph, and microfluidic techniques [34]. Despite being the standard technique for exosome separation because of its high processing capacity, ultra-speed centrifugation reveals lipoprotein contamination and high levels of protein aggregate in separated exosomes, which greatly affect their quantification and functional analysis using this technique [35]. Thus, tremendous efforts to explore the different physiochemical and biochemical properties of exosomes have been made to improve their separation.

Moreover, exosomes could be isolated from a cell culture medium by different methods to enhance their isolation outcome. Compared with the traditional isolation method using ultracentrifugation, the method based on tangential flow filtration increases the isolation yield of exosomes that originate from human umbilical cord MSCs (UCMSCs) 92.5 times [36]. The serum-depletion process has also yielded highly purified and clinically active exosomes from their cell culture medium [36]. By using a MSC surface marker (CD73), the purity of UCMSC-derived exosomes was reported to be enhanced 15.6 times using the process of depleting fetal bovine serum (FBS)-derived exosomes. Moreover, impurities resulting from FBS assessed by low-density lipoprotein-cholesterol levels were negligibly detected in the isolated exosomes. The angiogenic effects of highly purified UCMSC-derived exosomes were enhanced by approximately 71.4% *in vitro* using human coronary artery endothelial cells. Thus, the improvement of the purity and yield of MSC-derived exosomes affects their angiogenic and regenerative activities [36].

Exosomes could be regarded as mediators and biomarkers of various diseases including CVDs. The Osaka Acute Coronary Insufficiency Study revealed a positive correlation between the development of heart failure (HF) and levels of p53-responsive miRNAs inside EVs [37]. Levels of exosomal miRNA-9 and miRNA-124 could be used as promising biomarkers for the diagnosis of acute ischemic stroke, together with the serum IL-6 level, National Institutes of Health Stroke Scale (NIHSS) scores, and infarct sizes [38]. In addition, the exosomes isolated from monocrotaline-treated mice and patients with idiopathic pulmonary hypertension (PH) contained increased levels of miRNA-19b, miRNA-20a, miRNA-20b, and miRNA-145 [39]. Therefore, these exosomal miRNAs could be used for the diagnosis and prognosis of various CVDs. On the contrary, they could be used as therapies by acting as signaling agents that are capable of transferring specific peptides or miRNAs to target tissues or cells [40].

Surface markers such as CD9, CD63, and CD81 could characterize exosomes in addition to biogenesis-related specific marker proteins such as TSG101 and ALIX and heat-shock proteins (HSP60, HSP70, and HSP90). However, these markers are not exclusive to exosomes and could be revealed on other EVs [41]. Moreover, compared with their parents, exosomes could express different marker proteins. For example, the known endothelial marker CD144 is not present on exosomes derived from human umbilical vein endothelial cells (HUVECs) [40]. Moreover, the content of exosomes depends on the status of the parent cell [42]. The exosomes originated from chronic myelogenous leukemia were found to stimulate the release of IL-8 from BM stromal cells in addition to the enhancement of the progression of leukemia [43]. On the contrary, MSC-derived exosomes were found to alleviate symptoms in an experimental model of stroke by promoting angiogenesis, neurogenesis, and neurite remodeling [44].

Increasing evidence suggested that exosomes generated from stem cells exerted similar protective and reparative properties to their original cells. MSCs synthesize and secrete functional exosomes that are cholesterol-rich phospholipid vesicles, which afford direct protection of their RNA contents, specifically miRNAs against the action of RNase [45]. The therapeutic outcomes of exosomes and other EVs depend mainly on the delivery of peptides or non-coding RNAs, specifically miRNAs such as miRNA-21, miRNA-22, miRNA-24, and miRNA-29a, which have revealed cardioprotective roles in different diseases, along with other miRNAs [46–50].

## MSC-derived exosomes as therapy in different CVDs

MSC therapy has been studied in various CVDs, where its beneficial effects depend most probably on the paracrine secretion of bioactive molecules such as small non-coding miRNA. MSC release vesicles of various sizes loaded with miRNAs primarily for cell-to-cell communication. Research has shown that MSC-derived exosomes could accumulate in the ischemic myocardial tissue and regulate cell proliferation, apoptosis, inflammation, and angiogenesis [51, 52]. Exosomes can be used as drug delivery systems by being loaded with the required miRNAs to the target organ of interest, such as the heart. Hence, exosome-based cell-free therapy could be assessed as an alternative to cell-based therapy [53]. Importantly, Wang et al. identified miRNAs in human endometrium-derived MSC (EnMSC)-derived exosomes, particularly miRNA-21, as potential mediators of cardioprotection provided by EnMSC therapy. Thus, exosomes derived from other adult stem cells could be a subject of great attention as an alternative to cell-based therapy for CVDs (Table 1) [54].

**Table 1:** Studies on the efficacy of exosomes derived from MSCs and their roles in the treatment of various CVDs

No.	Disease	Title of study	Outcomes	References
1	Atherosclerosis	Mesenchymal stem-cell-derived exosomal miR-145 inhibits atherosclerosis by targeting JAM-A	Inhibition of JAM-A expression in plaque area and reduction of atherosclerosis plaque sizes	[55]
		Exosomes derived from mesenchymal stem cells attenuate the progression of atherosclerosis in ApoE−/− mice via miR-let7 mediated infiltration and polarization of M2 macrophage	Amelioration of atherosclerosis in ApoE−/− and promotion of M2 macrophage polarization in the plaque through miR-let7/HMGA2/NF-κB pathway in addition to suppression of macrophage infiltration via miR-let7/IGF2BP1/PTEN pathway in the plaque	[56]
		Mesenchymal stem cell-derived exosomal miR-21a-5p promotes M2 macrophage polarization and reduces macrophage infiltration to attenuate atherosclerosis	Promotion of macrophage polarization and reduction of macrophage infiltration by targeting KLF6 and ERK1/2 signaling pathways, thereby attenuating the development of atherosclerosis	[57]
2	Myocardial infarction	Bone marrow mesenchymal stem cell-derived exosomes protect against mmyocardial infarction by promoting autophagy	Decrease in apoptotic protease activating factor-1 and increase in autophagy-related protein 13 expression and thus inhibition of MI pathogenesis, possibly by regulating autophagy	[58]
		Combinatorial treatment of acute myocardial infarction using stem cells and their derived exosomes resulted in improved heart performance	Further improvement of cardiac function, reduction of infarct size, and increase in neovascularization through intramyocardial delivery of exosomes followed by MSCs transplantation in comparison to controls treated with exosomes or MSCs alone	[59]
		Mesenchymal stem cell-derived exosomes improve the microenvironment of infarcted myocardium contributing to angiogenesis and anti-inflammation	Stimulation of neovascularization, suppression of inflammatory response and improvement of heart function after ischemic injury	[60]
		Atorvastatin enhances the therapeutic efficacy of mesenchymal stem cells-derived exosomes in acute myocardial infarction via up-regulating long non-coding RNA H19	Enhancement of therapeutic efficacy through use of exosomes obtained from atorvastatin -pretreated MSCs possibly through promoting endothelial cell function	[61]
		Exosomal miR-25-3p from mesenchymal stem cells alleviates myocardial infarction by targeting pro-apoptotic proteins and EZH2	Cardioprotective effects *in vitro* and *in vivo* through inhibition of EZH2 or overexpression of miR-25-3p in cardiomyocytes	[62]
3	Heart failure	Adiponectin stimulates exosome release to enhance mesenchymal stem-cell-driven therapy of heart failure in mice	Enhancement of the therapeutic efficacy of MSCs through pharmacological or adenovirus-mediated genetic increase in plasma adiponectin and exosomes secretion	[63]
4	Peripheral arterial diseases	Mesenchymal stem cells release exosomes that transfer miRNAs to endothelial cells and promote angiogenesis	Enhancement of MSCs mediated angiogenesis and stem cell-to-endothelial cell communication through exosomal transfer of pro-angiogenic miRs (e.g., miR-30b)	[64]
		Exosomes derived from hypoxia-treated human adipose mesenchymal stem cells enhance angiogenesis through the PKA signaling pathway	Improved angiogenesis by activating the PKA signaling pathway and promoting the expression of VEGF	[65]
5	Stroke	Systemic administration of exosomes released from mesenchymal stromal cells promote functional recovery and neurovascular plasticity after stroke in rats	Improved functional recovery and enhanced neurite remodeling, neurogenesis, and angiogenesis	[44]
		Surface functionalized exosomes as targeted drug delivery vehicles for cerebral ischemia therapy	Strong suppression of the inflammatory response and cellular apoptosis in the lesion region of ischemic brain using curcumin loaded onto the engineered c(RGDyK) peptide-conjugated exosomes	[66]
6	Pulmonary hypertension	Mesenchymal stromal cell-derived exosomes improve pulmonary hypertension through inhibition of pulmonary vascular remodeling	Attenuation of right ventricular hypertrophy and pulmonary vascular remodeling in monocrotaline-induced pulmonary hypertension in rats	[67]
		Exosomes mediate the cytoprotective action of mesenchymal stromal cells on hypoxia-induced pulmonary hypertension	Pleiotropic protective effect on the lung using mesenchymal stromal cell–derived exosomes and inhibition of pulmonary hypertension through suppression of hyperproliferative pathways, including STAT3-mediated signaling induced by hypoxia	[68]
		Mesenchymal stromal cell exosomes ameliorate experimental bronchopulmonary dysplasia and restore lung function through macrophage immunomodulation	Alleviation of hyperoxia-induced bronchopulmonary dysplasia, improvement of lung function, decrease in fibrosis and pulmonary vascular remodeling, and amelioration of pulmonary hypertension	[69]
		Mesenchymal stromal cell-derived exosomes attenuate experimental pulmonary arterial hypertension	Reduction of right ventricular systolic pressure and right ventricular hypertrophy and suppression of the pulmonary vascular remodeling as well as the endothelial-mesenchymal transition process	[70]
		Intraperitoneal injection of MSC-derived exosomes prevent experimental bronchopulmonary dysplasia	Increase in blood vessel number and size in the lung through pro-angiogenic mechanisms and thus protection of the lung from bronchopulmonary dysplasia through anti-inflammatory and pro-angiogenic mechanisms	[71]

c(RGDyK), Cyclo(Arg-Gly-Asp-D-Tyr-Lys) peptide; *ERK*, Extracellular signal-regulated kinase; *EZH2*, Enhancer of zest homologue 2; *HMGA2*, High mobility group A protein 2; *IGF2BP1*, Insulin Like Growth Factor 2 MRNA Binding Protein 1; *JAM-A*, Junction adhesion molecule A; *KLF6*, Kruppel-like factor 6; *MI*, Myocardial infarction; *miRs*, microRNAs; *MSCs*, Mesenchymal stem cells; *NF-κB*, Nuclear factor kappa B; *PKA*, Protein kinase A; *PTEN*, Phosphatase and tensin homolog; *STAT3*, signal transducer and activator of transcription 3; *VEGF*, Vascular endothelial growth factor

### Atherosclerosis

Atherosclerosis is a chronic inflammatory disease of blood vessels that involves multiple processes such as lipid penetration, endothelial dysfunction, inflammatory response, and cell proliferation [55]. The development of atherosclerosis is initiated by endothelial dysfunction, where Zhang et al. [72] found that exosomes loaded with miRNA-155 promote atherosclerosis and induce endothelial injury. On the contrary, MSC-derived exosomes modulate the inflammatory response to atherosclerotic plaques, providing a potential way to prevent atherosclerosis [73]. MSC-derived exosomes also inhibited atherosclerosis in ApoE^−/−^ mice by preventing macrophage infiltration in addition to the promotion of M2 macrophage polarization in atherosclerotic plaques [56]. MSC-secreted exosomal miRNAs, which have been reported to regulate macrophage function, include miRNA-21-5p [63], miRNA-146a [64], miRNA-30a [65], and let-7 [66]. Besides, BM-derived MSC (BM-MSC) exosomal miRNA-133 can downregulate macrophage surface cholesterol efflux-related genes such as ATP-binding cassette subfamily A number 1 and ATP-binding cassette subfamily G number 1, resulting in interrupted cholesterol efflux and inhibiting foam cell formation [74]. MSC-derived exosomes also reverse the polarization status of macrophages by decreasing miRNA-182 expression and inhibiting the inflammation process [75]. Additionally, MSC-derived exosomal miRNA-145 can inhibit atherosclerosis by targeting junction adhesion molecule A (JAM-A) [55].

### Myocardial infarction (MI)

MI is a detrimental outcome of transient or persistent occlusion of coronary arteries with subsequent necrosis of the myocardium and loss of cardiomyocytes [76]. MSC transplantation into the infarcted region could improve cardiac function, a feature that could be enhanced by MSC modification through the upregulation of miRNA-1 that promotes MSC survival and their differentiation into cardiomyocytes [77]. As a compensatory ischemic signaling, exosomes released by the injured cardiomyocytes can transfer peptides and miRNAs to distant organs such as the BM. Moreover, exosomes from progenitor cells derived from the BM are delivered again to ischemic or injured tissues to allow their repair and regeneration [78].

Interestingly, under ischemic conditions, MSC-derived exosomes are loaded with elevated miRNA-22 levels in the injured cardiomyocytes to prevent apoptosis by targeting Mecp2 [79]. These exosomes also preserve ATP levels, reduce oxidative stress in addition to the induction of phosphatidylinositol 3-kinase/protein kinase B (Akt) signaling, and thus enhance the viability of the myocardium and prevent adverse outcomes of remodeling after ischemia/reperfusion injury [80]. Under the influence of miRNA-182, MSC-derived exosomes could also alter the macrophage polarization status from M1 to M2 and thus prevent myocardial ischemia/reperfusion injury [75]. MSC-derived exosomes also enhanced regional blood flow recovery in the injured heart through the promotion of angiogenesis [23] besides the attenuation of the induced inflammatory status [60]. Moreover, human MSC-derived exosomes enhanced cardiac contractility and calcium-handling gene expression in human-engineered cardiac tissue *in vitro*, highlighting their beneficial effects on cardiac contractility [81].

Exosomes isolated from Akt-overexpressing human UCMSCs have shown improvement of cardiac regeneration and promotion of angiogenesis through the activation of platelet-derived growth factor D [82]. Furthermore, the ability of exosomes obtained from mouse BM-MSCs to repair infarcted myocardium was enhanced after exposure to hypoxia, which was attributed to miRNA-210 and neutral sphingomyelinase 2 activities [83]. Notably, the combined delivery of exosomes and BM-MSCs in a sequential manner effectively reduced the scar size and restored heart functions after acute MI (AMI) in rats compared with those treated with exosomes or MSCs alone [59]. Therefore, MSC-derived exosomes could be an effective therapy for the management of AMI.

### HF

Mortality and morbidity are still major concerns in patients with HF despite the significant advances in its prevention and therapies [58]. In HF, the heart cannot pump the required blood to meet the body’s needs. Adverse remodeling following MI is one of the important factors that contribute to HF development. Endothelial dysfunction, imbalanced angiogenesis, and inflammation also critically contributed to the progression of HF [84].

Cardiomyocyte-derived exosomes of healthy volunteers significantly promoted the proliferation and reduced the apoptosis of neonatal rat cardiomyocytes *in vitro*, whereas exosomes from patients with HF produced the opposite outcomes, which may be secondary to decreased exosomal miRNA-21-5p that activates phosphatase and tensin homolog and downregulates Akt phosphorylation in cardiomyocytes [85]. Matsumoto et al. proposed that exosome-derived miRNAs can be used as a prognostic tool for HF in patients with AMI and elevated levels of p53-responsive miRNAs (34a, 192, and 194)18 days after AMI among patients experiencing HF within 1 year [37]. Exosome-associated p53-responsive miRNAs may also predict left ventricular remodeling after AMI [37]. Similarly, compared with healthy volunteers, patients with HF show elevated levels of miRNA-21and miRNA-92 [86] and reduced levels of miRNA-425 and miRNA-744in serum exosomes [87]. Notably, all these miRNAs are usually positively correlated with fibrosis and thus could be used for the diagnosis of hypertrophic heart diseases in addition to being possible targets to prevent their progression to HF [88].

Importantly, a previous study demonstrated that genetic loss of Alix, an important factor for exosome biogenesis, markedly diminished the *in vivo* production of exosomes by MSCs, affecting their therapeutic outcomes in a HF model and implicating their importance in mediating actions of MSCs [63]. On the contrary, some factors such as adiponectin were found to stimulate biogenesis and secretion of exosomes by MSCs, thereby enhancing their therapeutic efficacy in HF [63]. Ju et al. also reported that cardiac MSC-derived exosomes improved cardiac functions by enhancing capillary density and cardiomyocyte proliferation in a mouse AMI model [89]. Through their enhanced antifibrotic activity, MSCs overexpressing adrenomedullin can further enhance heart functions in rats with HF [90]. Moreover, exosomes secreted by human embryonic stem cell-derived cardiovascular progenitors demonstrated comparable cardioprotective potential to their parent cells in an experimental model of post-infarction HF [91].

### Peripheral arterial diseases (PADs)

PADs are diseases affecting peripheral arteries and thus blood flow to the limbs [92]. This may cause claudication symptoms and affect patients’ quality of life [93]. PAD is likely to be an indication of atherosclerosis, affects blood flow in arteries, and lessens blood supply to legs and, sometimes, to arms [92]. Recent evidence has indicated the success of stem cell-derived exosome application in the management of PAD by promoting angiogenesis [94, 95].

Exosomes from induced pluripotent stem cells-derived MSCs endorsed angiogenesis after injection into the ischemic limbs of mice [96]. EVs isolated from BM-MSCs were also found to enhance the formation of blood vessels in the ischemic limb *in vivo* by enriching them with vascular endothelial growth factor (VEGF) protein and miRNA-210-3p that upregulated VEGFR1 and VEGFR2 expressions in endothelial cells [97, 98]. In addition, Du et al. boosted the angiogenic potential of MSC-derived exosomes with a nitric oxide-releasing polymer in hind limb ischemia in a murine model where higher levels of VEGF and miRNA-126 cargo promoted angiogenesis [99]. Furthermore, CD34+ cells release exosomes that transfer miRNA-126-3p into their target cells to downregulate the expression of Sprouty-related EVH1 domain containing 1 and upregulate the genes that promote angiogenesis [100].

### Stroke

As one of the well-known fatal diseases, mortality and long-term disability prevail in ischemic stroke [101]. In ischemic stroke, blood vessel occlusion leads to brain ischemia with subsequent neuronal damage [102]. MSCs have shown great potential in the treatment of ischemic stroke through the release of biomolecules that induce angiogenesis [103], anti-apoptosis [104], and immunomodulation [105]. However, the difficulty to deliver MSCs limits their clinical application in ischemic stroke after intravenous administration because of the large diameters of MSCs (15–40 μm) where they are mostly captured by pulmonary capillaries, in addition topoor survival in inflammatory and hypoxic conditions of ischemic lesions [106]. The limitations of MSC therapy could be overcome by MSC-derived exosomes, as the pulmonary capillaries can permit nanosized exosomes, avoiding their accumulation in the lungs following systemic administration [107].

MSC-derived exosomes have been evaluated as therapies for the management of ischemic stroke [66, 108] where MSC-derived exosomes reveal more or less similar outcomes to those of MSCs. In an experimental model of transient middle cerebral artery occlusion, intravenous administration of MSC-derived exosomes stimulated the recovery of neuronal function through the induction of neurogenesis and neurite outgrowth and remodeling[44]. Other studies have revealed that MSC-derived exosomes exhibit nearly similar angiogenic, immunomodulatory, and neuroprotective potentials to MSCs [109, 110]. Interestingly, the efficacy of exosomes was enhanced by the glucose and oxygen deprivation of their treated MSCs, which could be related to the upregulation of some functional proteins in the derived exosomes [111, 112]. Excitingly, MSC-derived exosomes enhanced motor function recovery in a stroke model in primates [113]. These derived exosomes not only act by reducing neuroinflammation and shifting of the microglia into restorative functions [114], but by hampering injury-related hyperexcitability and restoring excitatory–inhibitory balance [115].

### PH

PH is a complicated pathological condition that is associated with the remodeling of pulmonary vessels, hypertrophy of the right ventricle, and subsequently its failure [116, 117]. No fully effective therapies are available for the management of vascular remodeling in PH. Exosomes isolated experimentally from monocrotaline-treated mice or clinically from patients with idiopathic pulmonary arterial hypertension showed upregulated levels of some miRNAs, such as miRNA-19b, miRNA-20a, miRNA-20b, and miRNA-145. On the contrary, MSC-derived exosomes were enriched with antiproliferative and anti-inflammatory miRNAs, namely, miRNA-34a, miRNA-122, miRNA-124, and miRNA-127. These assessments highlight the significant role of miRNAs in the prognosis or prevention of this disease [39]. Interestingly, the administration of exosomes from the conditioned media of BM-MSCs was demonstrated to reverse hypoxia-induced pulmonary fibrosis, leading to the full improvement of PH and right ventricular hypertrophy, whereas exosome-depleted media did not affect hypoxia-induced PH [68, 118]. Additionally, repeated administrations of MSC-derived exosomes were more effective than single administration in the reversal of semaxinib/hypoxia-induced PH in a rat model [119].

Several studies have shown that intravenous delivery of MSC-derived exosomes can inhibit PH vascular remodeling in different ways [39, 68, 119]. MSC-derived exosomes could alleviate PH through the reversal of pulmonary vascular remodeling and inhibition of its associated hyperproliferative pathways, including signal transducer and activator of transcription 3 (STAT3)-mediated signaling [68]. MSC-derived exosomes could also inhibit the proliferation of pulmonary arterial smooth muscle cells and apoptosis of hypoxia-induced pulmonary arterial endothelial cells through the upregulation of the expression of wnt5a and regulation of the RhoA and GSK3β/β-catenin signaling pathway [67]. MSC-derived exosomes attenuated PH in an experimental model of bronchopulmonary dysplasia through VEGF signaling with increased expression of VEGF/VEGFR2 [71]. Furthermore, MSC-derived exosomes may be enriched with protective miRNAs such as miRNA-483 [120] and miR-21-5p [121]. The overexpression of miR-483 in endothelial cells inhibits inflammatory and fibrogenic responses and leads to ameliorated PH phenotypes in rats [120]. MSC-derived exosomes alleviate ischemia/reperfusion injury in mouse lung by transporting anti-apoptotic miR-21-5p [121]. Importantly, intravenous administration of MSC-derived exosomes was found to upregulate miRNA-204 expression in the lungs, with the attenuation of the increased right ventricular systolic pressure in an experimental model of hypoxia-induced PH [68].

## Enhancement of the therapeutic effects of MSC-derived exosomes

**Fig. 2:**
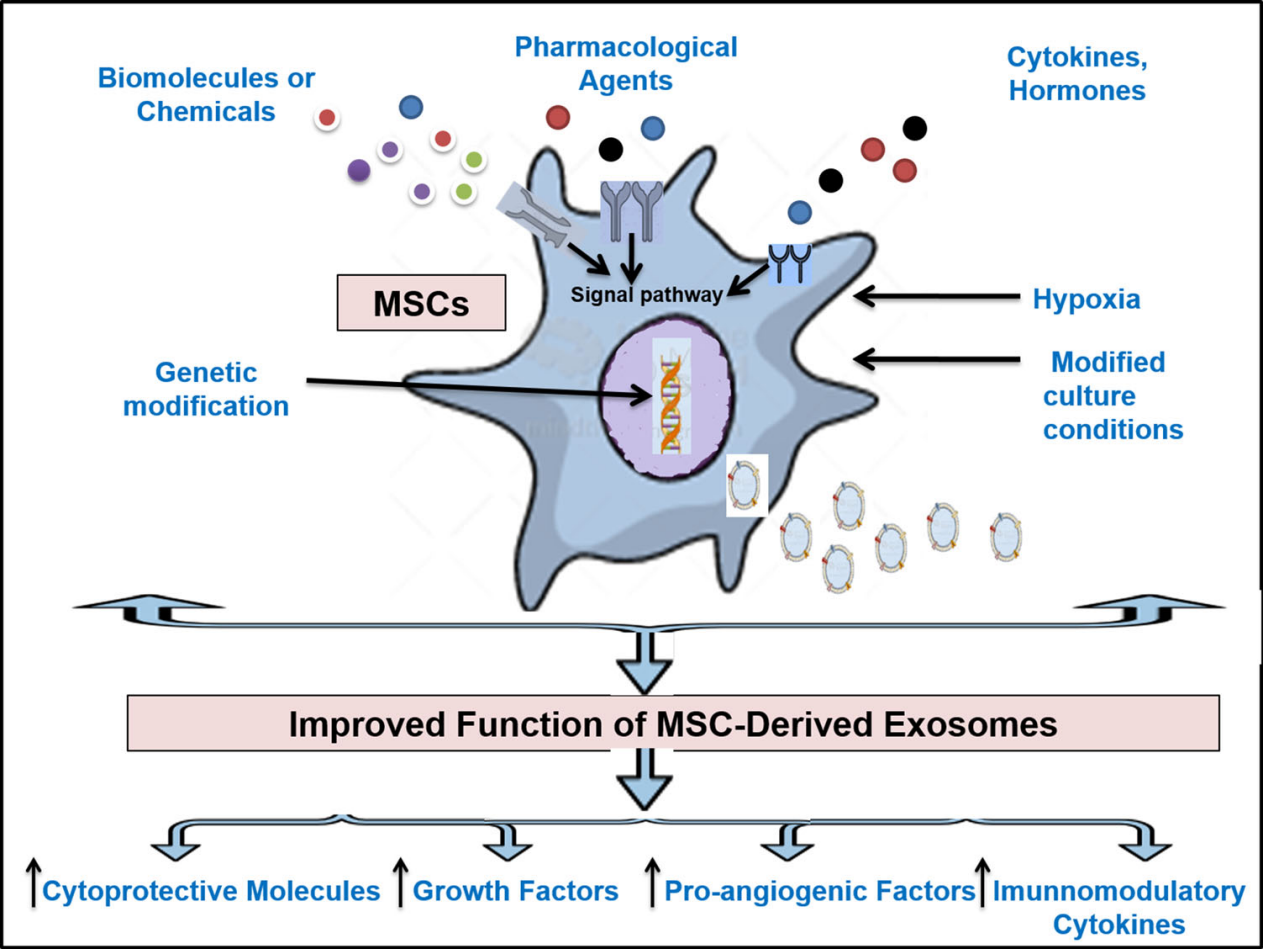
Different mechanisms for the improvement of the functions of MSC-derived exosomes

MSC-derived exosomes have been revealed as future candidates for cell-free therapy that could be used in the management of various diseases. However, a major challenge of their clinical application includes the maintenance of their efficacy and stability over time after their systemic transplantation [122]. As the characteristics of exosomes vary depending on the status of MSCs from which they are derived, exposure of MSCs to different external stimuli such as preconditioning with cytokines, hypoxia, and chemicals has enhanced the immunomodulatory and regenerative effects of their derived exosomes [123]. Moreover, genetic and cell surface modification of MSC-derived exosomes can modulate their therapeutic efficacy [124]. Different mechanisms could be used to improve functions of MSC-derived exosomes (Fig. 2).

### Preconditioning strategies

#### Increasing exosome production using different culture conditions

Although MSC-derived exosomes represent a promising candidate for cardiac regeneration and repair, their low level of production from routine culture conditions limits their therapeutic efficacy. Alterations of the characteristics of MSC-derived exosomes and their components are caused by the use of different culture conditions. For example, the culture of MSCs with the serum from the blood of mice with middle cerebral artery occlusion afforded more neuroprotection than that obtained from culture of MSCs with normal serum due to the remarkably upregulated expression of miRNA-20a in the released exosomes [125].

Importantly, exosomes from different microenvironments may have a focus on a specified cell type. For instance, neuron-derived EVs preferentially target cells in the nervous system [126]. However, further evaluation is needed to determine whether co-culturing of MSCs with the required cell type (e.g.,cardiomyocytes) can improve the efficacy of the released exosomes to target the same cell type. The production of MSC-derived exosomes could be also increased using 3D culture techniques. For example, the use of a 3D porous scaffold structure instead of 2D surfaces in plastic plates enhances the production MSC-derived exosomes and their therapeutic efficacy [127]. Microcarriers and bioreactors could be used for MSC expansions on large scale and thus increasing the amounts of their released exosomes [128]. Additionally, the use of osmotic stress such as vesiculation buffers containing chloride salts can enhance the production of EVs from targeted cells [129]. The usage of 1- to 2-μm pore polymer filters was found to improve the production of exosomes by extruding cells [130]. Furthermore, the amount of EVs produced can be enhanced by their treatment with cytochalasin B [131]. Similarly, MSC-derived exosomes treated with cytochalasin B have the same angiogenic potential to their parent MSCs [132].

#### Hypoxic preconditioning of MSCs

Hypoxic or ischemic preconditioning is a widely accepted approach for priming MSCs and MSC-derived exosomes. Several studies have demonstrated that exosomes from hypoxia-primed MSCs promote higher angiogenic potential relative to exosomes from MSCs cultured undernormoxic conditions [133, 134]. Salomon et al. reported that exosomes from hypoxia-primed placental MSCs increased the migration and angiogenic tube formation of placental microvascular endothelial cells [135]. In addition, hypoxia-primed MSCs produced exosomes that attenuated inflammation and elevated fat survival after grafting [136] with upregulated levels of angiopoietin-1, epidermal growth factor (EGF), and fibroblast growth factor proteins [134]. Vesicles produced by hypoxia-conditioned MSCs also induced protein kinase A signaling, vascular endothelial growth factor expression, and thus their angiogenic potential *in vitro* [137].

MSCs are enriched with miRNAs, which may promote cardiomyocyte survival during hypoxia [138]. Ischemic preconditioning induces BM-MSC survival by upregulating miRNA-210 expression, which targets caspase-8-associated protein [139]. Hypoxic preconditioning of BM-MSC exosomes could also inhibit cardiomyocyte apoptosis after AMI by upregulating miRNA-24 [140]. In addition, exosomes with overexpressed hypoxia-inducible factor 1-alpha were associated with elevated levels of proangiogenic factors that promoted neovessel development and prevented fibrosis in a rat model of MI [141]. The actions of exosomes from hypoxia-primed MSCs could be related to the transferred miRNA-210, which improves the angiogenic and anti-apoptotic functions of HUVECs, resulting in enhanced vascularization and survival rate [142]. These preconditioned exosomes also directly suppress GSK3β expression through miRNA-26a, resulting in the observed cardioprotective effects after MI [143].

#### Pharmacological preconditioning of MSCs

Preconditioning of MSCs with pharmacological agents may be considered an effective approach to enhance their efficacy. All these preconditioning techniques prevented apoptosis of transplanted MSCs *in vivo* and led to the improvement of their function [144, 145]. In general, these effects could be due to their enhanced angiogenic and antifibrotic potential. Preconditioning with several pharmacological agents [144–146] revealed a link between the enhanced effects and the increased levels of VEGF, fibroblast growth factor-2, or HGF and Akt signaling pathway activation. After their preconditioning with Buyang Huanwu decoction, MSC-derived EVs were demonstrated to increase VEGF levels and thus prevent brain injury in a rat model of cerebral ischemia [147]. Atorvastatin was found to enhance the immunomodulatory potential of MSCs by preventing inflammatory cell infiltration and thus the levels of pro-inflammatory cytokines *via* a C-X-C chemokine receptor type 4 (CXCR-4)-dependent mechanism. These effects would reduce the infarct size and improve functional recovery in a rat model of stroke [148]. Thus, paracrine actions of MSCs and their therapeutic outcomes are affected by their surrounding microenvironment even with minor changes in the culture conditions or changes associated with injured tissue microenviroment [149], and this would ultimately affect the actions of their derived exosomes.

#### Biomolecular or chemical preconditioning of MSCs

Biomolecules such as growth factors or others (i.e., angiotensin-II, EGF, glial cell line-derived neurotrophic factor, IGF-1, and stromal-derived factor 1 (SDF-1) and tumor necrosis factor α) were revealed to enhance the paracrine functions of MSCs and their regenerative capacity. Preconditioning of MSCs with interferon-gamma (IFN-γ) stimulates the IFN-primed MSCs to produce exosomes that are enriched with anti-inflammatory proteins, neuroprotective proteins, and anti-inflammatory RNAs [132, 150, 151].

Other biomolecules have been evaluated as preconditioning agents where exosomes derived from thrombin-primed MSCs revealed the improvement of proliferation, migration, and tube formation by HUVECs *in vitro* and in severe neonatal ischemic encephalopathy *in vivo* [152]. Interestingly, preconditioning of MSCs with traditional Chinese medicine that is used in the management of acute coronary syndrome improved the function of derived exosomes through the downregulation of the expression of the H3K27 demethylase UTX and hence the enhancement of cardiomyocyte proliferation [153]. Exosomes from NO-preconditioned MSCs also increasedthe levels of VEGF and miRNA-126, which could be proposed as a novel mechanism contributing to the increased angiogenic capacity of these exosomes [99]. In addition, chitosan hydrogel could enhance the retention and stability of exosomes and augment their therapeutic and angiogenic effects in a model of hind limb ischemia [122].

### Genetically modified MSC-derived exosomes

The use of genetically modified MSCs is another approach to enhance the efficacy of exosomes by the knockdown or overexpression of certain RNAs. For instance, transfecting MSCs with a recombinant adenovirus with the Akt gene would upregulate the levels of platelet-derived growth factor D (PDGF-D) produced in their derived exosomes [82] and thus enhance their angiogenic, regenerative, and reparative potential.

Exosomes from GATA-4-overexpressing MSCs were demonstrated to improve cardiac function by decreasing apoptosis and increasing the number of newly formed blood vessels in a mouse model of MI [154]. MSCs with overexpressed GATA-4 are enriched with anti-apoptotic miRNA-19a, which induces the extracellular signal-regulated kinase (ERK) and Akt signaling pathways [138]. Likewise, MSCs with overexpressed CXCR4 were shown to release exosomes with enhanced tube formation by HUVECs and significant cardioprotective effects through the activation of Akt signaling in a rat model of MI [155]. In another study, MSCs overexpressing Akt released exosomes with enhanced angiogenic functions *in vitro* and improved regenerative potential *in vivo* by activating PDGF-D in a rat model of acute MI [82]. Exosomes derived from MSCs with overexpressed indoleamine 2,3-dioxygenase were found to increase the Tregs/CD8 + T-cell ratio and hence inhibited the production of pro-inflammatory cytokines and enhanced the immune tolerance in a rat model of cardiac allografts [156]. Furthermore, exosomes derived from MSCs with overexpressed SDF-1were shown to enhance cardiac repair through the inhibition of apoptosis and promotion of endothelial microvessel regeneration in a mouse model of MI [157].

Similarly, the overexpression of miRNAs in MSCs can enhance the efficacy of exosomes. MSCs overexpressing miRNA-133 produced exosomes that decreased inflammation and fibrosis in a rat model of AMI [158]. MSCs with overexpressed miRNA-146 and miRNA-93-5p also released exosomes that inhibited myocardial damage in a rat model of MI. These exosomes also inhibited *in vitro* the inflammatory cytokine expression and autophagy in hypoxic H9c2 cells [159]. Similarly, MSCs with overexpressed miRNA-126 released exosomes that promoted microvascular generation and suppressed the expression of hypoxia-induced inflammation factors *in vitro* in addition to the inhibition of cardiac inflammation and fibrosis *in vivo* [160]. Thus, genetically modified MSC-derived exosomes could be an effective way to enhance their efficacy in regenerative medicine.

## Use of exosomes as targeted drug delivery systems

Exosomes exhibit unique characteristics that make them an ideal vehicle for drug delivery. Their existence in all biological fluids increases their tolerance in the body when used as drug delivery vehicles [161]. Exosomes could be also loaded with specific drugs or biomolecules for the development of specific therapeutic outcomes. Therapeutic loading of exosomes could be performed with the required agents before or after being isolated from biological fluids [162]. Pasucci et al. revealed that paclitaxel (PTX)-loaded MSC-derived exosomes isolated after the incubation of PTX with MSCs exhibited anti-tumor activity [163]. Another feature that supports the use of exosomes as vehicles for drug delivery includes their ability to cross the blood–brain barrier [164] and produce their therapeutic effect [165, 166]. The intravenous administration of exosomes conjugated with RGDyK can target the ischemic lesion in a model of middle cerebral artery occlusion in mice. Furthermore, the loading of these exosomes with curcumin suppressed inflammation and apoptosis in the ischemic region [66]. All these factors make exosomes an ideal vehicle for drug delivery in various diseases including CVDs.

Importantly, the systemic administration of exogenous exosomes for therapeutic purposes is technically challenging and subjected to macrophage-mediated phagocytosis in the liver where the vast majority of exosomes accumulate, leading to the rapid decrease in their circulation levels after intravenous administration [167–169]. This implies the need to generate higher doses of exosomes to meet the clinical requirements, which is usually limited by the number of cellular sources available for secreting sufficient amounts of exosomes [170].

Modifying the surface of exosomes using different pre-and post-isolation methods could be useful to control their pharmacokinetics by elevating their circulating half-life, increasing their bioavailability to target tissues, and improving their therapeutic efficacy [170]. For example, coating their surface using PEGylation method prevents them from being aggregated or subjected to phagocytosis, prolonging their half-life and enhancing their blood circulation time sixfold in comparison to unmodified exosomes [170, 171]. Several labeling methods such as bioluminescence emitted from luciferase could be used to evaluate the pharmacokinetics of exosomes and their biodistribution [172]. However, all structural modifications of exosomes should be tested for their safety and efficacy in well-designed *in vivo* models.

Recent evidence showed that, unlike other vectors that have been used for gene delivery, exosomes could be used to carry different contents including miRNAs to adjacent or distant targeted cells [173, 174] to mediate the crosstalk among cell types and provide reliable way to overcome the inefficient and nonspecific delivery [175, 176]. The challenge is how to effectively load these exosomes with required biomaterials without affecting their structure or ability to interact with target cells. Electroporation is a commonly applied technique that has been used to add anti-inflammatory compounds to exosomes from macrophages with a loading efficacy of approximately 20% and was successfully evaluated by reducing inflammation in acute peritonitis and atherosclerosis development [177]. Transfection techniques also have been used to load exosomes with the desired cargo, such as miRNAsor proteins. Recent findings have revealed that exosomes loaded with syndecan-1 ameliorated pulmonary edema through the reduction of pro-inflammatory cytokines (IL-1β, tumor necrosis factor-α, and IL-6) [178]. Additionally, exosomes loaded with miR-21-5p, a miRNA that is downregulated in HF, exhibited good regenerative potential in MI unlike that derived from cardiac stromal cells in patients with HF [85]. Therefore, exosomes could be used as potential vectors of required biomolecules, which could fundamentally change the current therapeutic management of CVDs in the future.

## Limitations and future challenges

The use of stem cell-derived exosomes has many advantages compared with the transplantation of parent stem cells, where the induction of IFN-γ can increase the risk of immune rejection following the MSC transplantation [179]. On the contrary, the derived exosomes revealed low immunogenicity and negligible risk of tumor development [180]. MSC-derived exosomes are more easy and stable to store than MSCs [57] in addition to their relatively lower cost production [181]. Exosomes show no obvious adverse reactions, such as fever and allergic or hemolytic reactions [182]. Additionally, exosomes can produce stronger therapeutic effects, as they can cross capillaries without plugging because they are much smaller than their parent cells [183]. Repeated administrations of BM-MSC-derived exosomes did not show any harmful immune reactions or toxicity in mice as evaluated by immunotyping of various tissues and histological examinations [128, 184]. Thus, exosomes could be more efficacious than their parent cells after systemic administration by the recapitulation of the beneficial effects of their parent cells and overcoming their limitations [52].

Despite these significant advantages, the application of exosomes has several limitations [24] including inconvenient and time-wasting procedure used for extraction using the classic high-speed centrifugation in addition to the limitation of the amount of exosomes produced. Besides, several harmful and unwanted components could be present in the exosomes, which should be modified and removed. Furthermore, only few exosomes reach the target area after systemic administration, which could greatly restrict their efficiency [168]. Thus, several challenges should be addressed to harness the full therapeutic potential of exosomes.

Several considerations should be evaluated before the clinical applications of exosomes. First, more accurate isolation methods should be adopted other than that based on their size, as different vesicle sizes may reflect only different components in the isolated exosomes. For example, their isolation may depend on the epitopes expressed by the donor cell. Additionally, MSC-derived exosomes may be contaminated by exosomes of various cell types, as they are usually isolated from blood or culture media. Thus, extracting the exosomes from multivesicular endosomes before being released may help get purified exosomes and overcome the problem of contamination. It is also important to understand the mechanism of exosome dynamic secretion and their uptake at the tissue level in addition to the establishment of a well-defined method for single-exosome analysis rather than the whole-exosome population. Finally, recent evidence demonstrates that MSC-derived exosomes may exhibit different therapeutic outcomes according to the variations of their components in different microenvironments. For instance, MSCs may release exosomes that exhibit immunomodulatory actions after MI in the pro-inflammatory phase and then exosomes that exert pro-angiogenic actions in the reparative and regenerative phases [52]. Taken together, continuous attempts are required to understand the exact mechanisms of the therapeutic actions of exosomes and hence the possibility of their modification or enhancement. More studies are also needed to evaluate the best route of administration of exosomes, their optimal dose, and their pharmacokinetics and biodistribution inside the body. Importantly, the engineered exosomes should be tested for their efficacy, immunogenicity, and safety in general [183–185].

## Conclusion

CVDs are a major cause of death worldwide; thus, continuous efforts have beenmade to search for novel therapeutic strategies to trigger the repair and regeneration of injured tissues. In experimental studies, exosomes have emerged over the past decade as an alternative to cell-based therapy in various CVDs with promising and beneficial outcomes concerning neovascular, anti-apoptotic, anti-remodeling, and anti-inflammatory actions. Exosomes could be used both as biomarkers or mediators of various diseases in addition to their management by targeting injured areas with specific peptides or miRNAs or other factors. Despite continuous efforts to enhance the isolation and production yield of MSC-derived exosomes and efficacy and stability over time after *in vivo* transplantation, more investigations and approaches are required to overcome the limitations of their use before their clinical application.
